# Supplementary material for: Modeling glioblastoma heterogeneity as a dynamic network of cell states
Source: Mol Syst Biol. 2021 Sep 16;17(9):e10105. doi: 10.15252/msb.202010105 (PMC8444284; doi:10.15252/msb.202010105)
Supplement: Supplementary file 5 — Source Data for Figure 3 [file MSB-17-e10105-s001.zip › Figure3A_sourcedata/GSEA_3065/hallmarks_state2.GseaPreranked.1623416269049/HALLMARK_DNA_REPAIR.html]

Details for gene set HALLMARK\_DNA\_REPAIR[GSEA]

|  || Dataset | state2 |
| Phenotype | NoPhenotypeAvailable |
| Upregulated in class | na\_pos |
| GeneSet | HALLMARK\_DNA\_REPAIR |
| Enrichment Score (ES) | 0.6161328 |
| Normalized Enrichment Score (NES) | 2.1313505 |
| Nominal p-value | 0.0 |
| FDR q-value | 0.0 |
| FWER p-Value | 0.0 |
Table: GSEA Results Summary

  

Fig 1: Enrichment plot: HALLMARK\_DNA\_REPAIR      
 Profile of the Running ES Score & Positions of GeneSet Members on the Rank Ordered List

  

| PROBE | GENE SYMBOL | GENE\_TITLE | RANK IN GENE LIST | RANK METRIC SCORE | RUNNING ES | CORE ENRICHMENT || 1 | TYMS |  |  | 5 | 0.792 | 0.0537 | Yes |
| 2 | PCNA |  |  | 16 | 0.595 | 0.0933 | Yes |
| 3 | DUT |  |  | 24 | 0.552 | 0.1303 | Yes |
| 4 | RPA3 |  |  | 36 | 0.511 | 0.1641 | Yes |
| 5 | ZWINT |  |  | 45 | 0.488 | 0.1966 | Yes |
| 6 | RFC2 |  |  | 53 | 0.460 | 0.2274 | Yes |
| 7 | ALYREF |  |  | 91 | 0.382 | 0.2495 | Yes |
| 8 | FEN1 |  |  | 107 | 0.371 | 0.2733 | Yes |
| 9 | POLD3 |  |  | 120 | 0.347 | 0.2957 | Yes |
| 10 | RFC4 |  |  | 126 | 0.339 | 0.3184 | Yes |
| 11 | NME1 |  |  | 214 | 0.274 | 0.3278 | Yes |
| 12 | POLR2F |  |  | 253 | 0.250 | 0.3408 | Yes |
| 13 | LIG1 |  |  | 259 | 0.246 | 0.3571 | Yes |
| 14 | SAC3D1 |  |  | 263 | 0.246 | 0.3736 | Yes |
| 15 | NME4 |  |  | 295 | 0.234 | 0.3863 | Yes |
| 16 | RFC5 |  |  | 297 | 0.233 | 0.4021 | Yes |
| 17 | POLR2I |  |  | 315 | 0.227 | 0.4158 | Yes |
| 18 | SSRP1 |  |  | 319 | 0.226 | 0.4310 | Yes |
| 19 | POLA2 |  |  | 366 | 0.212 | 0.4406 | Yes |
| 20 | RPA2 |  |  | 367 | 0.212 | 0.4551 | Yes |
| 21 | POLR2J |  |  | 417 | 0.196 | 0.4633 | Yes |
| 22 | GPX4 |  |  | 425 | 0.194 | 0.4758 | Yes |
| 23 | COX17 |  |  | 445 | 0.191 | 0.4868 | Yes |
| 24 | RBX1 |  |  | 494 | 0.182 | 0.4941 | Yes |
| 25 | ADRM1 |  |  | 508 | 0.179 | 0.5050 | Yes |
| 26 | POLR2E |  |  | 524 | 0.177 | 0.5155 | Yes |
| 27 | POLR2K |  |  | 545 | 0.173 | 0.5252 | Yes |
| 28 | HPRT1 |  |  | 573 | 0.168 | 0.5337 | Yes |
| 29 | IMPDH2 |  |  | 575 | 0.167 | 0.5451 | Yes |
| 30 | TAF10 |  |  | 586 | 0.165 | 0.5553 | Yes |
| 31 | POLE4 |  |  | 597 | 0.164 | 0.5654 | Yes |
| 32 | RFC3 |  |  | 617 | 0.161 | 0.5744 | Yes |
| 33 | PRIM1 |  |  | 660 | 0.155 | 0.5805 | Yes |
| 34 | GTF2A2 |  |  | 662 | 0.155 | 0.5909 | Yes |
| 35 | POLA1 |  |  | 752 | 0.143 | 0.5912 | Yes |
| 36 | NELFE |  |  | 755 | 0.143 | 0.6007 | Yes |
| 37 | POLR2H |  |  | 787 | 0.140 | 0.6070 | Yes |
| 38 | DGUOK |  |  | 845 | 0.133 | 0.6100 | Yes |
| 39 | RALA |  |  | 1015 | 0.118 | 0.5999 | Yes |
| 40 | ERCC1 |  |  | 1065 | 0.114 | 0.6024 | Yes |
| 41 | POLR2D |  |  | 1137 | 0.109 | 0.6023 | Yes |
| 42 | UMPS |  |  | 1145 | 0.109 | 0.6089 | Yes |
| 43 | TAF12 |  |  | 1148 | 0.108 | 0.6161 | Yes |
| 44 | GUK1 |  |  | 1276 | 0.100 | 0.6093 | No |
| 45 | MPC2 |  |  | 1335 | 0.096 | 0.6096 | No |
| 46 | NUDT21 |  |  | 1379 | 0.093 | 0.6114 | No |
| 47 | POLD1 |  |  | 1452 | 0.089 | 0.6098 | No |
| 48 | AAAS |  |  | 1453 | 0.089 | 0.6159 | No |
| 49 | NELFB |  |  | 1628 | 0.080 | 0.6027 | No |
| 50 | SF3A3 |  |  | 1767 | 0.074 | 0.5929 | No |
| 51 | EDF1 |  |  | 1775 | 0.073 | 0.5972 | No |
| 52 | GTF2F1 |  |  | 1785 | 0.073 | 0.6012 | No |
| 53 | UPF3B |  |  | 1888 | 0.069 | 0.5950 | No |
| 54 | TSG101 |  |  | 1925 | 0.067 | 0.5957 | No |
| 55 | POLR2C |  |  | 2007 | 0.064 | 0.5914 | No |
| 56 | EIF1B |  |  | 2035 | 0.063 | 0.5928 | No |
| 57 | ITPA |  |  | 2048 | 0.063 | 0.5959 | No |
| 58 | GTF2H3 |  |  | 2129 | 0.060 | 0.5914 | No |
| 59 | SMAD5 |  |  | 2156 | 0.059 | 0.5926 | No |
| 60 | TAF6 |  |  | 2179 | 0.058 | 0.5942 | No |
| 61 | SUPT4H1 |  |  | 2307 | 0.053 | 0.5842 | No |
| 62 | MRPL40 |  |  | 2340 | 0.052 | 0.5844 | No |
| 63 | ARL6IP1 |  |  | 2421 | 0.050 | 0.5792 | No |
| 64 | APRT |  |  | 2426 | 0.050 | 0.5822 | No |
| 65 | RAE1 |  |  | 2454 | 0.049 | 0.5827 | No |
| 66 | CETN2 |  |  | 2466 | 0.049 | 0.5848 | No |
| 67 | POLR2G |  |  | 2491 | 0.048 | 0.5855 | No |
| 68 | ELOA |  |  | 2601 | 0.045 | 0.5769 | No |
| 69 | NT5C3A |  |  | 2728 | 0.041 | 0.5661 | No |
| 70 | POLR3GL |  |  | 2924 | 0.036 | 0.5476 | No |
| 71 | SNAPC5 |  |  | 2955 | 0.035 | 0.5468 | No |
| 72 | ADA |  |  | 3009 | 0.034 | 0.5434 | No |
| 73 | TAF9 |  |  | 3040 | 0.033 | 0.5424 | No |
| 74 | SDCBP |  |  | 3058 | 0.033 | 0.5428 | No |
| 75 | USP11 |  |  | 3142 | 0.031 | 0.5360 | No |
| 76 | SUPT5H |  |  | 3217 | 0.029 | 0.5301 | No |
| 77 | NELFCD |  |  | 3233 | 0.029 | 0.5304 | No |
| 78 | ERCC8 |  |  | 3317 | 0.027 | 0.5234 | No |
| 79 | GTF2H5 |  |  | 3473 | 0.024 | 0.5084 | No |
| 80 | TP53 |  |  | 3495 | 0.024 | 0.5078 | No |
| 81 | GTF2B |  |  | 3537 | 0.023 | 0.5050 | No |
| 82 | POLR2A |  |  | 3680 | 0.021 | 0.4912 | No |
| 83 | POLR3C |  |  | 3717 | 0.020 | 0.4887 | No |
| 84 | ERCC3 |  |  | 3877 | 0.018 | 0.4728 | No |
| 85 | TARBP2 |  |  | 3970 | 0.016 | 0.4640 | No |
| 86 | POM121 |  |  | 4110 | 0.014 | 0.4500 | No |
| 87 | POLH |  |  | 4128 | 0.014 | 0.4491 | No |
| 88 | SURF1 |  |  | 4221 | 0.012 | 0.4400 | No |
| 89 | POLR1D |  |  | 4259 | 0.011 | 0.4368 | No |
| 90 | NT5C |  |  | 4470 | 0.008 | 0.4148 | No |
| 91 | ELL |  |  | 4514 | 0.007 | 0.4106 | No |
| 92 | SRSF6 |  |  | 4555 | 0.006 | 0.4068 | No |
| 93 | NFX1 |  |  | 4706 | 0.004 | 0.3909 | No |
| 94 | DCTN4 |  |  | 4815 | 0.002 | 0.3795 | No |
| 95 | SNAPC4 |  |  | 4823 | 0.002 | 0.3789 | No |
| 96 | CSTF3 |  |  | 4868 | 0.002 | 0.3743 | No |
| 97 | POLR1C |  |  | 5107 | -0.002 | 0.3488 | No |
| 98 | NCBP2 |  |  | 5108 | -0.002 | 0.3490 | No |
| 99 | DDB1 |  |  | 5130 | -0.002 | 0.3468 | No |
| 100 | CANT1 |  |  | 5151 | -0.002 | 0.3449 | No |
| 101 | XPC |  |  | 5262 | -0.004 | 0.3333 | No |
| 102 | BRF2 |  |  | 5307 | -0.005 | 0.3289 | No |
| 103 | PDE4B |  |  | 5498 | -0.008 | 0.3091 | No |
| 104 | GTF2H1 |  |  | 5636 | -0.011 | 0.2951 | No |
| 105 | AK3 |  |  | 5911 | -0.015 | 0.2667 | No |
| 106 | REV3L |  |  | 5959 | -0.016 | 0.2628 | No |
| 107 | GMPR2 |  |  | 5997 | -0.017 | 0.2599 | No |
| 108 | TAF13 |  |  | 6079 | -0.018 | 0.2524 | No |
| 109 | GTF3C5 |  |  | 6083 | -0.018 | 0.2533 | No |
| 110 | VPS37D |  |  | 6217 | -0.020 | 0.2404 | No |
| 111 | VPS28 |  |  | 6507 | -0.025 | 0.2111 | No |
| 112 | VPS37B |  |  | 6609 | -0.027 | 0.2022 | No |
| 113 | ERCC2 |  |  | 6652 | -0.028 | 0.1996 | No |
| 114 | POLB |  |  | 6884 | -0.034 | 0.1771 | No |
| 115 | ERCC4 |  |  | 7017 | -0.037 | 0.1654 | No |
| 116 | GSDME |  |  | 7223 | -0.043 | 0.1463 | No |
| 117 | NUDT9 |  |  | 7274 | -0.044 | 0.1440 | No |
| 118 | RNMT |  |  | 7302 | -0.045 | 0.1441 | No |
| 119 | MPG |  |  | 7337 | -0.046 | 0.1436 | No |
| 120 | DDB2 |  |  | 7419 | -0.048 | 0.1381 | No |
| 121 | TMED2 |  |  | 7420 | -0.048 | 0.1414 | No |
| 122 | TK2 |  |  | 7488 | -0.050 | 0.1376 | No |
| 123 | TAF1C |  |  | 7713 | -0.057 | 0.1175 | No |
| 124 | RRM2B |  |  | 8015 | -0.068 | 0.0898 | No |
| 125 | BCAM |  |  | 8026 | -0.068 | 0.0934 | No |
| 126 | ADCY6 |  |  | 8196 | -0.077 | 0.0805 | No |
| 127 | DGCR8 |  |  | 8245 | -0.079 | 0.0808 | No |
| 128 | DAD1 |  |  | 8847 | -0.128 | 0.0250 | No |
| 129 | BCAP31 |  |  | 9017 | -0.152 | 0.0172 | No |
| 130 | NME3 |  |  | 9204 | -0.192 | 0.0104 | No |
| 131 | SEC61A1 |  |  | 9261 | -0.217 | 0.0192 | No |
Table: GSEA details [plain text format]

  

Fig 2: HALLMARK\_DNA\_REPAIR: Random ES distribution      
 Gene set null distribution of ES for **HALLMARK\_DNA\_REPAIR**
